# Clarifying the role of the resist–accept–direct framework in supporting resource management planning processes

**DOI:** 10.1111/cobi.70062

**Published:** 2025-05-20

**Authors:** Gregor W. Schuurman, Wylie Carr, Cat Hawkins Hoffman, David J. Lawrence, Brian W. Miller, Erik A. Beever, Jean Brennan, Katherine R. Clifford, Scott Covington, Shelley D. Crausbay, Amanda E. Cravens, John Gross, Linh Hoang, Stephen T. Jackson, Abraham J. Miller‐Rushing, Wendy Morrison, Elizabeth A. Nelson, Robin O'Malley, Jay O. Peterson, Mark T. Porath, Karen Prentice, Joel H. Reynolds, Suresh A. Sethi, Helen R. Sofaer, Jennifer L. Wilkening

**Affiliations:** ^1^ Climate Change Response Program National Park Service Fort Collins Colorado USA; ^2^ U.S. Geological Survey North Central Climate Adaptation Science Center Boulder Colorado USA; ^3^ U.S. Geological Survey Northern Rocky Mountain Science Center Bozeman Montana USA; ^4^ Department of Ecology Montana State University Bozeman Montana USA; ^5^ Climate Adaptation Landscape Partnership Auberry California USA; ^6^ Western Water Assessment University of Colorado Boulder Colorado USA; ^7^ Science Applications in the Pacific Region U.S. Fish and Wildlife Service Portland Oregon USA; ^8^ USDA Forest Service Fort Collins Colorado USA; ^9^ U.S. Geological Survey Forest and Rangeland Ecosystem Science Center Corvallis Oregon USA; ^10^ Mountain Planning Service Group USDA Forest Service Missoula Montana USA; ^11^ U.S. Geological Survey (Emeritus) National Climate Adaptation Science Center Tucson Arizona USA; ^12^ Acadia National Park National Park Service Bar Harbor Maine USA; ^13^ Office of Sustainable Fisheries NOAA National Marine Fisheries Service Silver Spring Maryland USA; ^14^ Parks Canada Vancouver British Columbia Canada; ^15^ Robin O'Malley LLC Fort Collins Colorado USA; ^16^ Office of Science and Technology NOAA Fisheries Silver Spring Maryland USA; ^17^ Ecological Services U.S. Fish and Wildlife Service Wood River Nebraska USA; ^18^ USDOI Bureau of Land Management Fort Collins Colorado USA; ^19^ Solutions Consulting, LLC Fort Collins Colorado USA; ^20^ Aquatic Research & Environmental Assessment Center Brooklyn College Brooklyn New York USA; ^21^ U.S. Geological Survey Pacific Island Ecosystems Research Center Hawaii National Park Hawaiʻi USA; ^22^ National Wildlife Refuge System, Natural Resource Program Center U.S. Fish and Wildlife Service Fort Collins Colorado USA

**Keywords:** Adaptive management, climate change adaptation, communication, conservation, ecological transformation, planning

## INTRODUCTION

The resist–accept–direct (RAD) framework was developed by and for conservationists, resource managers, and climate change adaptation practitioners and scientists to foster strategic and collaborative thinking about responses to anthropogenic ecological change (Lynch et al., 2021; Schuurman et al., 2022, 2020; Thompson et al., 2021). Prevailing management approaches, which emphasize managing for ecosystem stationarity and maintaining historical ecological conditions or dynamics (e.g., Landres et al., 1999), are increasingly inadequate in this time of rapid, directional change (Jackson, 2021; Schuurman et al., 2022). Resisting anthropogenic environmental change has been the traditional approach in the resource management community. However, thinking beyond persistence alone is critical, given that preservation of all ecological components and processes in any given place will not be possible as the environment in which they developed transforms. This change in thinking constitutes a paradigm shift that calls for new tools and approaches, and the RAD framework is gaining traction in conservation and resource management agencies (e.g., the United States Department of the Interior [USDOI, 2021], the National Park Service [NPS, 2021, 2024], Australia's Parks Victoria Board [PVB, 2022], and South African National Parks [van Wilgen‐Bredenkamp et al., 2024]).

The RAD framework helps managers navigate transformative ecological change by defining a broad decision space that encompasses managing for persistence to managing for change and includes resisting (R) ecological trajectories moving away from historical or natural conditions; consciously accepting (A) such change; and directing (D) ecological trajectories toward preferred new conditions. By fostering deliberative thinking about options that include accepting and directing change, RAD is intended to help managers expand their thinking beyond traditional resistance approaches. By providing a structured way to consider a wide, even novel, set of options, RAD supports a necessary shift in perspective, helping managers respond to often‐rapid ecological transformations.

The RAD framework is also designed to promote collaboration and communication among diverse partners, stakeholders, and rights holders in planning and decision‐making processes. The framework's simple, 3‐part framing focuses on manager action and establishes a common, policy‐neutral vocabulary that can foster joint or complementary actions across landscapes and jurisdictions and coherency in climate‐informed goals (Magness et al., 2022; Schuurman et al., 2022; Ward et al., 2023). In sum, RAD is intended to be a simple framework that promotes exploration of a wider decision space while providing straightforward, intuitive concepts and vocabulary that foster interdisciplinary collaboration and communication in adaptation planning processes.

## RAD FRAMEWORK'S RELATIONSHIP WITH CONSERVATION AND RESOURCE MANAGEMENT PLANNING PROCESSES

**FIGURE 1:**
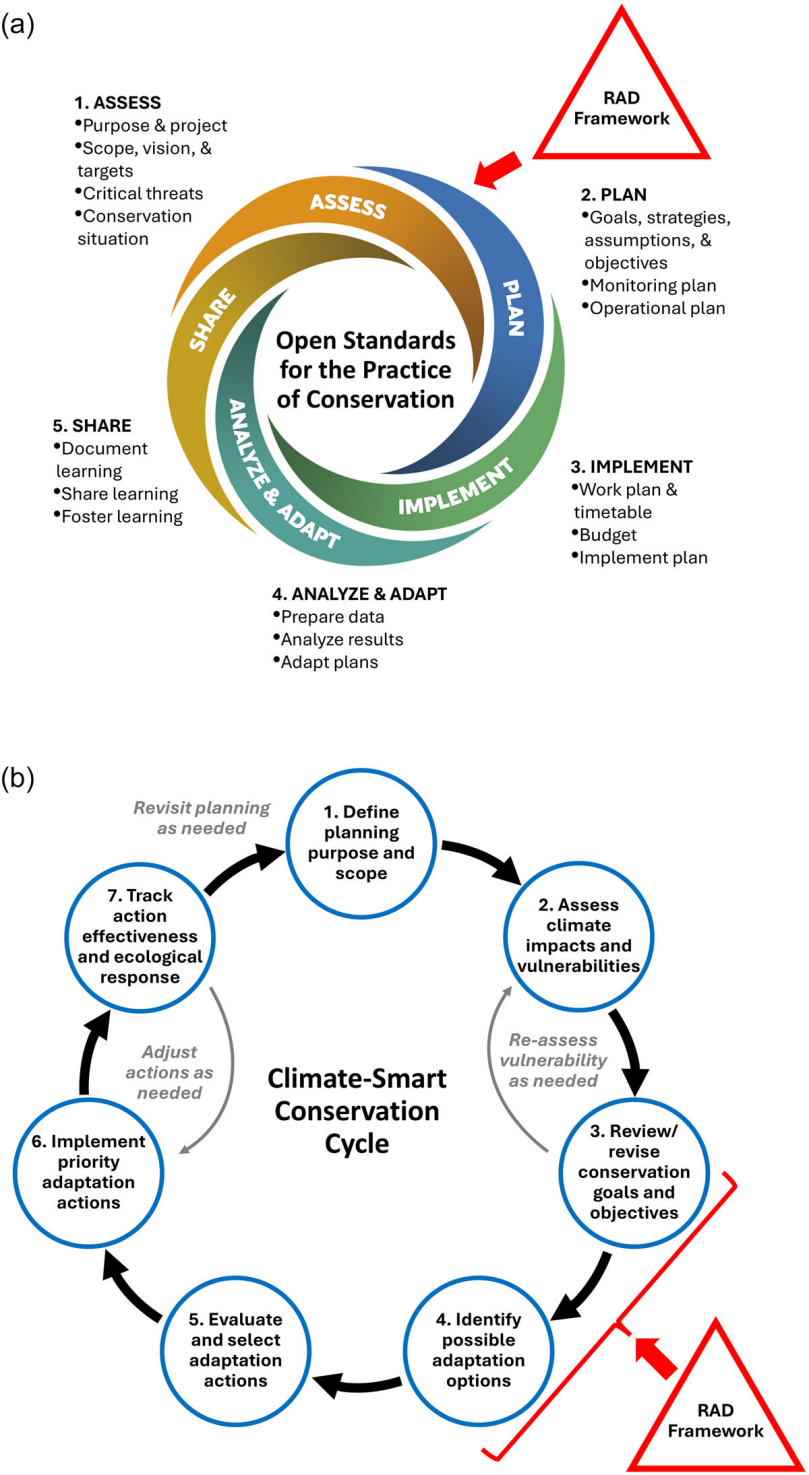
The resist–accept–direct (RAD) framework, which supports existing adaptive‐management‐based planning and decision‐making processes, principally during the design step, as illustrated for (a) Open Standards for the Practice of Conservation (CMP, 2020) and (b) the Climate‐Smart Conservation Cycle (Stein et al., 2014). Figures adapted from CMP (2020) and Stein et al. (2014).

Although intended to be a modest framework for expanding the management decision space, RAD is sometimes conflated with a stand‐alone planning and decision‐making process. However, by itself, RAD is not a complete planning process. Instead, the framework—developed by multiple U.S. federal agencies and partners in recognition that each organization has its own mission, policies, and planning approaches—was intentionally designed for integration into a broad range of planning and decision‐making processes (Figure 1). The NPS, for example, uses Planning for a Changing Climate (NPS, 2021), a 6‐step climate change adaptation process, whereas the U.S. Forest Service uses a 5‐step process in their *Adaptation Workbook* (Swanston & Janowiak, 2012; Swanston et al., 2016) for site‐level planning. Other organizations use similar guidance and processes, such as Climate‐Smart Conservation (Stein et al., 2014), the PrOACT decision model (Hammond et al., 1998), the ACT framework (Cross et al., 2012), the European Adaptation Support Tool (Pringle et al., 2015), and Open Standards for the Practice of Conservation (CMP, 2020). All are consistent with the theory and practice of adaptive management (Williams, 2011), a “special case of structured decision‐making, applicable when the decision is iterated over time or space” (Lyons et al., 2008, p. 1684). Lynch et al. (2022) describe 3 case studies that highlight RAD application in a generic adaptive management context.

The key to effective RAD‐based resource management is understanding that the RAD framework is designed to fit within—rather than to supplant—an adaptive management process (e.g., Schuurman et al., 2024). Thus, downstream stages in cyclical planning and decision‐making processes (e.g., considering trade‐offs, selecting options, implementing actions) occur after the RAD framework has been used to develop adaptation options (Figure 1).

## CONCLUSION

The RAD framework supports a fundamental shift in how managers clarify intent and generate options for resource stewardship in a changing, warming world. As a straightforward and intuitive tool, the framework can be readily integrated in existing planning processes to explore the full spectrum of management options. Further, by providing a “common language” (Schuurman et al., 2022, p. 26), the intentional simplicity of RAD promotes collaboration and clear communication among organizations with different mandates, policies, and planning and decision‐making processes, thus promoting adaptation from local to landscape scales.
